# A Time-to-Digital Converter for Low-Power Consumption Single Slope Analog-to-Digital Converters in a High-Speed CMOS Image Sensor

**DOI:** 10.3390/mi15050578

**Published:** 2024-04-27

**Authors:** Ziyi Li, Zhiyuan Gao

**Affiliations:** School of Microelectronics, Tianjin University, 92 Weijin Road, Tianjin 300072, China

**Keywords:** time-to-digital converters, image sensors, low power consumption, high speed

## Abstract

To reduce the power consumption of a TDC in high-speed applications, a TDC architecture applied to SS ADC is proposed to reduce redundant counting. This structure can remove the identical part between two rows of pixel signals in a CMOS image sensor by adjusting the *start* and *stop* signal of the TDC, which will reduce the number of flipping of D flip-flops in the TDC. This structure requires the simultaneous readout of two rows of pixels in the high-speed CMOS image sensor. In the 110 nm CMOS process, simulation results show that the designed 5-bit TDC achieves an effective number of bits (ENOB) at 4.72 bits and a figure-of-merit (FOM) at 104.7–162.3 fJ/step, with a power consumption ranging from 60 µW to 93 µW. Compared with traditional counting methods, the proposed TDC can reduce counting power consumption by 30%.

## 1. Introduction

The surge in high-speed computing tasks—ranging from target tracking and machine vision to scientific exploration—has escalated the need for advanced CMOS image sensors characterized by superior frame rates and responsiveness. CMOS image sensors generally integrate analog-to-digital converters (ADCs) for multi-bit quantization. Single Slope (SS) ADCs integrated with TDCs, emerge as a promising solution for enhancing CMOS image sensors’ speed without compromising image quality [1,2,3]. Nonetheless, the conventional delay chain-based TDCs face challenges in escalating power consumption and physical size with the expansion of quantization levels [4].

A time-to-digital converter (TDC) can measure the time difference between two input events or pulse widths. There are many architectures for high-resolution TDCs, including a vernier-based delay chain TDC [5], a time amplifier-based TDC [6,7], and a gated-ring oscillator (GRO) TDC [8]. A purely digital TDC based on a delay chain offers high throughput and a straightforward structure [9], making it a popular choice with diverse applications.

The use of gated delay technology to reduce power consumption can be found in Ref. [10]. A power management circuit that generates a gated delay in a two-step TDC is designed, which can make the gated delay signal keep low at TDC non-sampling times; the delay chain is closed at this time, so as to reduce power consumption. In Ref. [11], the authors use histogram memory instead of an analog counter to achieve the 3300-fold power reduction. The analog counters and a timing generator in each pixel are reconfigured to an SS ADC with a self-referenced ramp mitigating nonuniformities from counters. A low-power TDC structure is described in Ref. [12], which generates the digital code of the input delay time based on the difference between two consecutive delay time samples. This structure takes advantage of the continuity in analog signals over time to reduce the circuit activity. However, due to the introduction of a time amplifier, the speed of this TDC is limited. In this work, a TDC structure applied to CMOS image sensors is proposed by taking advantage of the spatial continuity of analog signals. This structure enables the simultaneous quantization of two rows of pixel signals from the CMOS image sensor. This dual-row readout approach is particularly well suited for high-speed CMOS image sensors with SS ADCs. Eliminating redundant data quantized between the two rows of signals reduces the D flip-flop flipping frequency in the delay chain, resulting in a more than 30% reduction in power consumption during counting. The TDC counting method proposed can reduce power consumption in most scenarios but is not applicable when the input image is an extreme case, such as an image that contains black and white stripes. Since the area of TDC will increase exponentially with the increase of quantization bits, and high quantization bits will restrict the speed characteristics, the design of TDC proposed in this paper is 5-bit. It is more suitable for high-speed image sensors rather than high imaging accuracy.

The remainder of this paper is organized as follows: In Section 2, we present the working principle of traditional SS ADC based on TDC and the power analysis of TDC, which demonstrates the advantage of reducing the redundant counting scheme of TDC. Then we describe the proposed TDC that quantifies the difference between two adjacent rows of pixel signals. In Section 3, we present a modeling analysis of the proposed TDC counting method and demonstrate its power reduction capability. In Section 4, we present the simulation results of the proposed TDC in a 110 nm process. Finally, the conclusions are drawn in Section 5.

## 2. Proposed Structure

### 2.1. Traditional Structure

**Figure 1:**
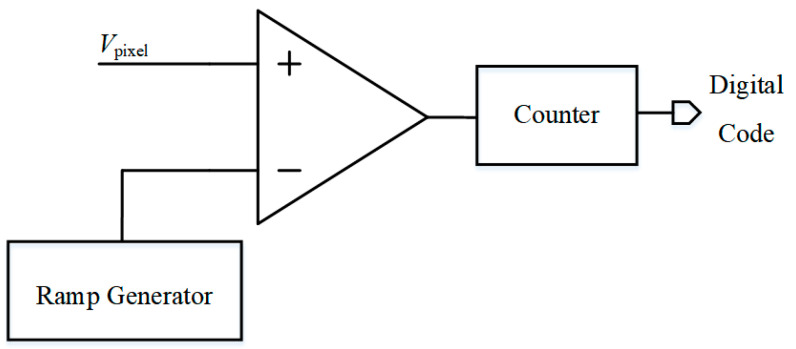
Structure of traditional SS ADC.

The structure of traditional Single Slope ADC is shown in Figure 1, which comprises a comparator, ramp generator, and counter. *V*_pixel_ is the output signal of the pixel in the CMOS image sensor. The ramp generator initiates a gradually decreasing voltage signal, with the counter tallying from the onset of this ramp. The pixel output is then compared to this slope voltage, yielding the SS ADC’s quantization result after decoding.

The TDC-based SS ADC converts the counter into a TDC. In this mode of operation, the SS ADC compares the pixel signal with the ramp signal. If the pixel signal voltage exceeds the ramp voltage, the comparator flips, and its output is transmitted to the TDC. The TDC then measures the time from the comparator flip to the end of the clock cycle.

**Figure 2:**
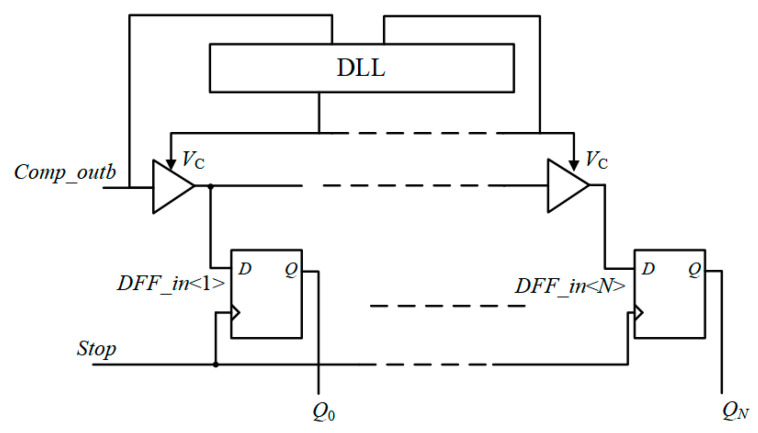
TDC structure−based on delay chain.

The traditional TDC structure for CMOS image sensors is shown in Figure 2, which is mainly composed of a delayed phase-locked loop, delay chain, and D flip-flop. *DFF_in*<*N*> represents the input of the *N*-th D flip-flop, which is the output of the *N*-th delay unit. *Comp_outb* represents the inversion signal of the comparator. The pixel output is transmitted to the column level for comparison with the reference ramp, and the point in time at which the comparator flips is transmitted to the delay-locked loop (DLL) delay chain. In order to complete *n*-bit quantization, 2*^n^* delay units are required. The rising edge of the comparator flip is delayed by each delay unit with the same time accuracy, and the output of each delay unit is connected to the signal input of the D flip-flop. The rising edge of the next clock cycle will be sent to the clock input of the D flip-flop as a *stop* signal. The clock signal will sample the signal input from the D flip-flop and read the output of the delay unit into the D flip-flop. Then the D flip-flop is used as a shift register, the data in the D flip-flop are passed to the decoder, and the final *N*-bit quantization result is obtained.

### 2.2. Power Consumption Analysis of TDC

The main power consumption of a TDC comes from the power consumption of the delay unit and the D flip-flop, where the delay unit ensures the accuracy of the delay time, and its current magnitude is determined by the *V*_c_ provided by the DLL. The power consumption of the CMOS circuit is

(1) $$P_{T}=P_{\mathrm{st}}+P_{\mathrm{dyn}}$$

where *P*_T_ is the total power consumption, *P*_st_ is the static power consumption, and *P*_dyn_ is the dynamic power consumption. Static power consumption is mainly caused by a drain current, and static power consumption is

(2) $$P_{\mathrm{st}}=I_{D}\times V_{\mathrm{DD}}$$

where, *I*_D_ is the drain current and *V*_DD_ is the power supply voltage. Dynamic power consumption is mainly caused by the charge and discharge of the circuit to the node capacitor. Each charge (or discharge) of a node capacitor *C* will lead to the dynamic power consumption of 1/2*CV*_DD_^2^. Therefore, the average static power consumption of a node *i* in the circuit during the whole working time can be expressed as [13]

(3) $$P_{\mathrm{dyn}}=\frac{1}{2}C_{i}V_{\mathrm{DD}}^{2}E_{\mathrm{sw}}(i)f_{\mathrm{clk}}$$

where *f*_clk_ is the clock frequency and *E*_sw_ is the switching activity, which represents the average number of jumps in the node *i* signal in each clock cycle. Therefore, the more the number of signal jumps in the D flip-flop, the higher its dynamic power consumption, resulting in a higher total power consumption.

**Figure 3:**
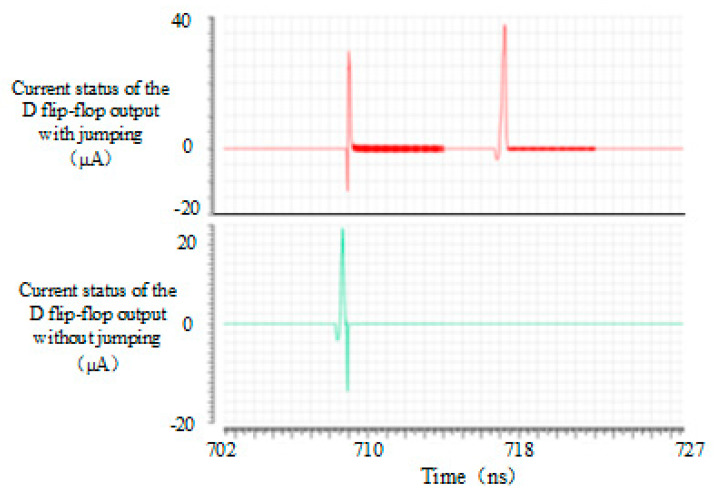
D current status of the flip−flop output with or without jumping.

In the simulation process, it can be found that when the signal input of the D flip-flop is kept at a low level, its power consumption level is far less than that when the signal input has a high level. Simulation of the D flip-flop was carried out. The simulation results are as shown in Figure 3, respectively. The current condition of the output in the D flip-flop becomes high-level and the current condition of the input and output of the D flip-flop is kept low-level. The average current when the output remained low was 32.88 nA. The average current when the output changed was 217.2 nA. It could be seen that the average current in D flip-flop would be significantly increased by flipping. The power consumption of D flip-flop was mainly the power consumption of its flipping. Therefore, if an advanced counting method is adopted to reduce the flipping times of the D flip-flop in TDC, low power consumption can be achieved.

**Figure 4:**
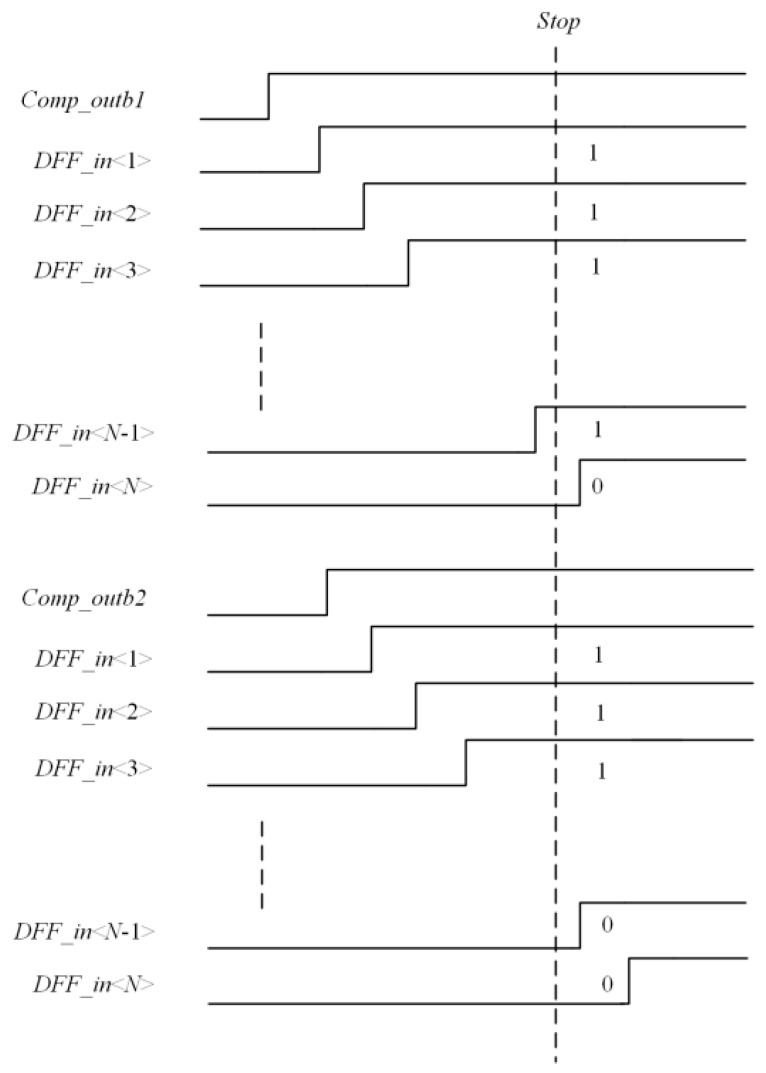
The output of the delay chain in the quantization process.

The output of the delay chain in the quantization process is shown in Figure 4. Where *Comp_outb*1 and *Comp_outb*2 represent the inversion signal of the comparator in the first and second rows, respectively. When the *stop* signal arrives, each D flip-flop will sample the output of the delay unit and generate a thermometer code with one and zero separated as 111…00. Due to the continuity of the analog signal, the light intensity information between the two adjacent rows of pixels is generally very close, so its quantization code value will also be very close. If a more simplified quantization method that quantizes the difference between two rows of pixel signals rather than the full signal can be adopted to eliminate the quantization of the same part between the two rows of signals, the quantification of redundant information will be removed. The number of output signals of one can be reduced; in other words, the number of flips of the D flip-flop can be reduced, which could reduce the counting power consumption.

The main source of ADC differential nonlinearity is the random variation in the delay time of the delay unit buffer caused by the inconsistency and mismatch of the device manufacturing. The greater the number of delay buffers, the greater the differential nonlinearity, which will cause a certain error. In addition, the D flip-flop must ensure that it has a narrow metastable width, which must be shorter than the delay period of the delay unit so as to reduce the TDC time measurement error.

### 2.3. Proposed TDC

**Figure 5:**
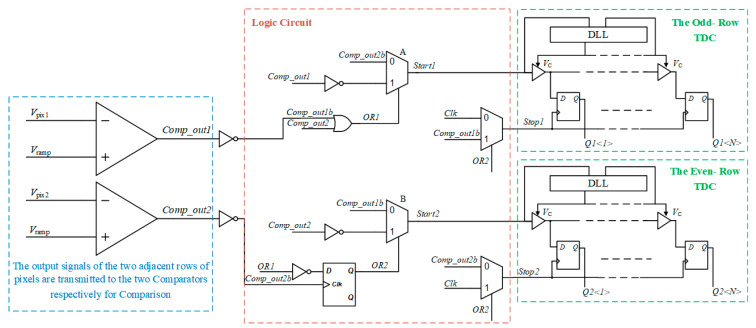
Improved column level readout architecture.

The improved column-level readout architecture is shown in Figure 5, where *start*1 and *stop*1 are the start and stop signals of the odd-line TDC, respectively, *start*2 and *stop*2 are the start and stop signals of the even-line TDC, respectively, and OR1 and OR2 are the outputs of the two OR gates, respectively. Since the output of two rows of pixels needs to be compared to quantify their difference, two column parallel buses need to be used for a simultaneous readout, and the output of pixels is sent to the column-level comparator for comparison with the reference slope voltage, respectively. When the output signals of the two rows of pixels are different, the flip time of the comparator will also be different. By sending the outputs of the two comparators into two sets of TDC for quantization at the same time, the flip time point of the comparator, i.e., the output signal of the pixel, can be encoded.

In high-speed image sensors, considering the RC delay in the row driver signal transmission line and column bus, the row driver signal is difficult to be established from the timing control driver circuit on both sides of the pixel array to the middlemost column pixel, and the pixel signal is difficult to be output from the middlemost row to the column level through the column bus during the row selection time.

Under the premise of not reducing the pixel array and frame rate, to achieve complete signal transmission within the row time, the mode of simultaneous readout of two column buses proposed in this work can be adopted. Each column pixel shares two column buses, and the pixel output of odd and even rows is transmitted to the column level, respectively. In this way, the timing control driver circuit can select two adjacent rows of pixels each time. The signal is selected and read at the same time; each column bus only needs to complete the selecting and reading of half of the pixel output in one frame time, and the row selection time can be doubled, which greatly alleviates the pressure of signal establishment in the row selection time. Therefore, the proposed TDC is very suitable for high-speed CMOS image sensors.

**Figure 6:**
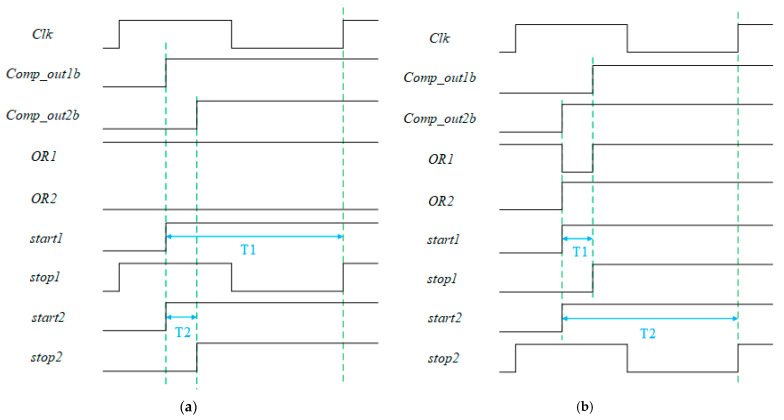
Timing of proposed TDC quantization. (**a**) Odd row signals arrive first. (**b**) Even row signals arrive first.

For TDC to quantify the difference between the output of two rows of pixels, it is necessary to quantify the time difference between the flip time points of the two rows of comparators. A series of logic circuits were designed before TDC, and the working sequence is shown in Figure 6, where *Comp_out1b* is the inverting signal for the odd-line comparator, *Comp_out2b* is the inverting signal for the even-line comparator, and T1 and T2 represent the length of time that the TDC of odd and even lines need to be quantized, respectively.

In the initial state disjunction gate output, *OR1* is high when *Comp_out*1, i.e., the first-row comparator, flip time point arrives earlier than *Comp_out*2, disjunction gate output remains high, the output of multiplexer A is the delay in the first-row comparator flip invert signal, and the signal which will be delayed by the delayed chain is sent to the *start* signal input of the first row TDC. At this time, the clock input of D flip-flop has not yet arrived, and its output is the default value after reset, i.e., the low level. The output of multiplexer B is also the delay in the first row in the comparator flipping invert signal, and the *start* signal of the two rows of TDC is the delay in the first row in the comparator flipping invert signal. After that, even if the second row in the comparator flipping time arrives, the value of *OR*1 remains unchanged. The output of the D flip-flop will also remain low; Since the output of the D flip-flop remains low, the *stop* signal of the first-row TDC is the rising edge of the next clock cycle, and the *stop* signal of the second-row TDC is the delay in the second-row comparator flip invert signal.

When *Comp_out*2 arrives before *Comp_out*1, the output *OR*1 of the disjunction gate will become low, the output of multiplexer A will be the delay in the second-row comparator flip invert signal, the output of the D flip-flop will also jump to a high level, and multiplexer B will select the delay in the second-row comparator flip invert signal. At this time, the *stop* signal of the second row in the TDC is the rising edge of the next clock cycle, and the *stop* signal of the first row in the TDC is the delay in the first row in the comparator flip invert signal.

It can be seen that the first arrived comparator flip signal will be fully quantized from the time of the flip time point to the rising edge of the next clock cycle, and the other row in the TDC completes the quantization of the time difference between the flip points of the two rows of comparators. Due to the spatial continuity of the analog signal, the difference is usually relatively small. Therefore, this quantization method can reduce the number of flips of the D flip-flop, thereby reducing the power consumption of the count. In addition, in traditional TDC-based SS ADCs, an edge detection circuit is usually needed to generate a *stop* signal, that is, the comparator flip signal is delayed, and the D flip-flop is used to sample the end of the clock cycle where TDC quantization is located, i.e., the rising edge of the next clock cycle. In the improved TDC, the *stop* signal of the faster row in the comparator flipping signal is the end of the whole quantization time, and the *stop* signal of the slower row in the comparator flipping signal is the *stop* signal of the other row in the TDC. Therefore, the improved TDC does not need the edge detection circuit to provide the *stop* signal, which can further save power consumption.

## 3. Modeling Analysis

The low-power counting method introduced above was modeled and analyzed. The counting model was used to quantify the image, and the gray value of the image was converted into the input signal for the pixels. The analog-to-digital conversion model was established according to the improved working mode of TDC, and the gray value of every two rows was compared.

**Figure 7:**
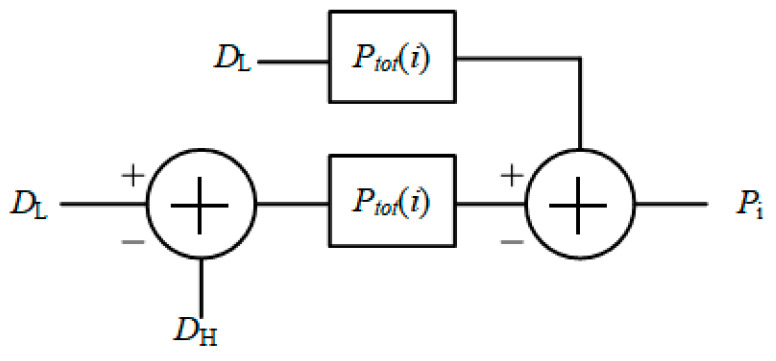
Counting model of TDC.

As shown in the Figure 7, *D*_H_ and *D*_L_ represent higher and lower pixel gray values, respectively. *P*_i_ represents the total power consumption of the improved structure. Data with a larger gray value is quantified as the difference in code values between the two rows. The quantized part of the code value represents the number of flips of the D flip-flop, and the unquantized part is multiplied by the static power consumption of the D flip-flop, plus the power consumption of each logical unit to obtain the total power consumption. The total power consumption is written as

(4) $$P_{\mathrm{tot}}(i)=i\cdot P_{d}+(2^{n}-i)\cdot P_{s}+P_{L}$$

where *P*_tot_(*i*) represents the total power consumption of a TDC, *i* is the number of D flip-flops with high output levels, *n* is the quantization bit, *P*_d_ and *P*_s_ represent, respectively, the flip power consumption and static power consumption of D-flip-flops, *P*_L_ represents the power consumption of other logic circuits.

**Figure 8:**
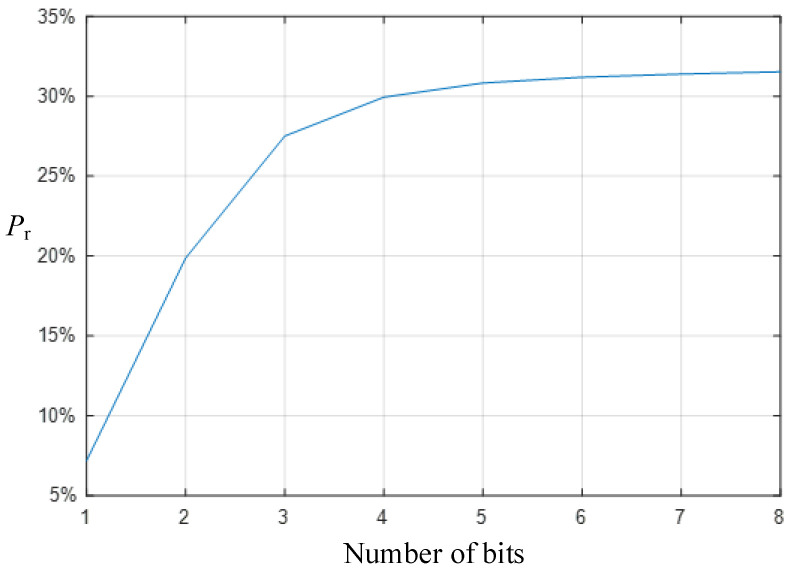
The power consumption of the improved counting model is reduced.

The power consumption reduction level of the improved counting mode is shown in Figure 8. The power consumption reduction level *P*_r_ is written as

(5) $$P_{r}=(1-\frac{P_{i}}{P_{\mathrm{tr}}})\times 100\%$$

where *P*_i_ represents the power consumption of the improved TDC, and *P*_tr_ represents the power consumption of the traditional structure.

The same part between the two rows of pixel signals is quantized only once, the number of flips of the D flip-flop is significantly reduced, and unnecessary redundant counts are removed. Compared with the traditional quantization method, the improved low-power counting method significantly reduces power consumption. The higher the quantization accuracy, the more obvious the power consumption reduction level. When the quantization bit is three, the low-power counting method can reduce the counting power consumption by more than 25%; when the quantization bit is further increased, the power consumption reduction degree tends to be stable, at about 32%.

With the increase of quantization bits, the effect of the TDC power consumption reduction proposed in this paper will be more significant. However, the number of delay units and D flip-flops in TDC is 2*^n^*, where *n* is the quantization bits of TDC. Therefore, with the increase of quantization bits, the number of delay units and D-flip-flops will increase exponentially and occupy a larger area; at the same time, under the condition of constant time precision, the length of the TDC delay chain determines the quantization time of TDC, and the increase in the delay unit will make the delay chain longer, and then increase the quantization time of the TDC. Therefore, in order to control the area of TDC and improve the quantization speed, the TDC proposed in this paper is designed to be 5-bit. The number of delay units and D flip-flops is 32.

## 4. Simulation Results

**Figure 9:**
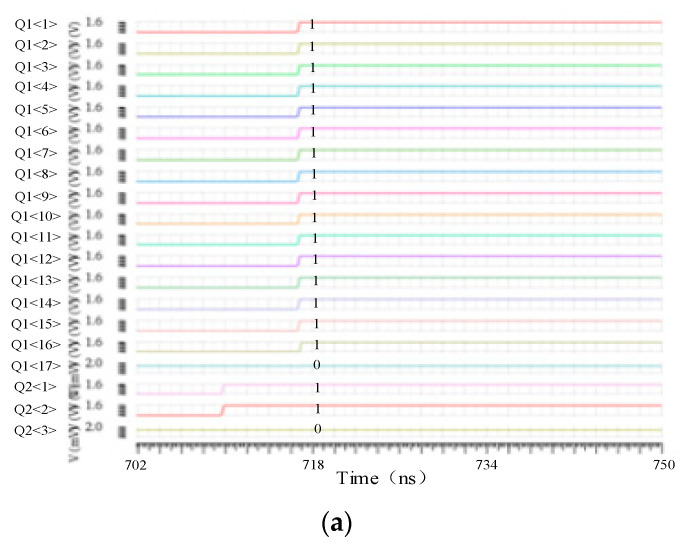

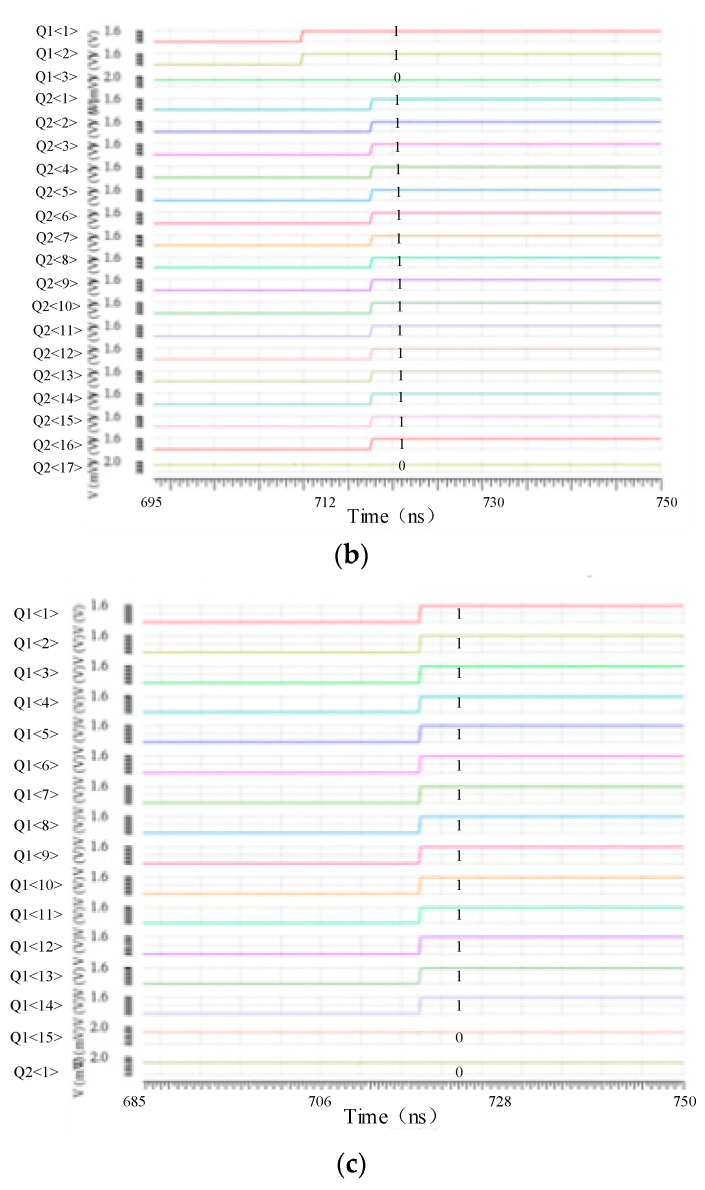
Functional simulation of TDC in different situations. (**a**) Odd row signals arrive first. (**b**) Even row signals arrive first. (**c**) The odd row signal and the even row signal arrive at the same time.

The improved TDC simulation results are shown in Figure 9a–c, respectively, when *Comp_out*1 signal arrives first, when *Comp_out*2 arrives first, and when *Comp_out*1 and *Comp_out*2 arrive simultaneously. The figure shows the result after transcoding the thermometer code of the TDC, where Q1 represents the output of the TDC in odd rows, Q2 represents the output of the TDC in even rows, and each flip indicates that the TDC outputs a “1”. It can be seen from the simulation results that the designed TDC can fully quantify the complement of the first arrived signal, and the other row in the TDC can quantify the difference between the two rows of signals.

However, in the process of reducing the timing accuracy of TDC, it was found that the multiplexer control signal at the input will pass through a D flip-flop before the *start* signal of the second row in the TDC enters, resulting in a 350 ps delay in the *start* signal. When the time accuracy is less than 1 ns, the delay effect is very significant. This will cause the TDC quantization to lose one number. In order to solve this problem, the *stop* signal in this case is delayed by the same means. The *stop* signal is delayed by the D flip-flop before the clock signal enters the TDC. It was proven that this method can effectively reduce the quantization error of the TDC.

When the proposed TDC structure is used as a fine quantization counter in a two-step SS ADC, if the two comparator flip points are in two different coarse quantization clock cycles, the number will be miscounted. Therefore, a judgment circuit or gate input was required to be the inverting output of the edge detection circuit, the *stop* signal of the current line and the output of the first D flip-flop. At the end of the coarse quantized clock cycle where the comparator flip point was reached first, in other words, when the rising edge of the next clock cycle arrived, if the *stop* signal of the current line is still low at this time, it means that the two comparators did not flip in the same coarse quantization period. In this case, the *start* signal of the line receives the output of the comparator, and the *stop* signal receives the output of the edge detection circuit. In this case, the *start* signal will change from a high level to a low level, and the output result of the D flip-flop is the complement. In order to reduce this situation as much as possible, the quantization number of the TDC can be as large as possible, which can make the coarse quantization clock cycle longer, and also alleviate the energy consumption problem caused by too high of a clock frequency to a certain extent.

**Figure 10:**
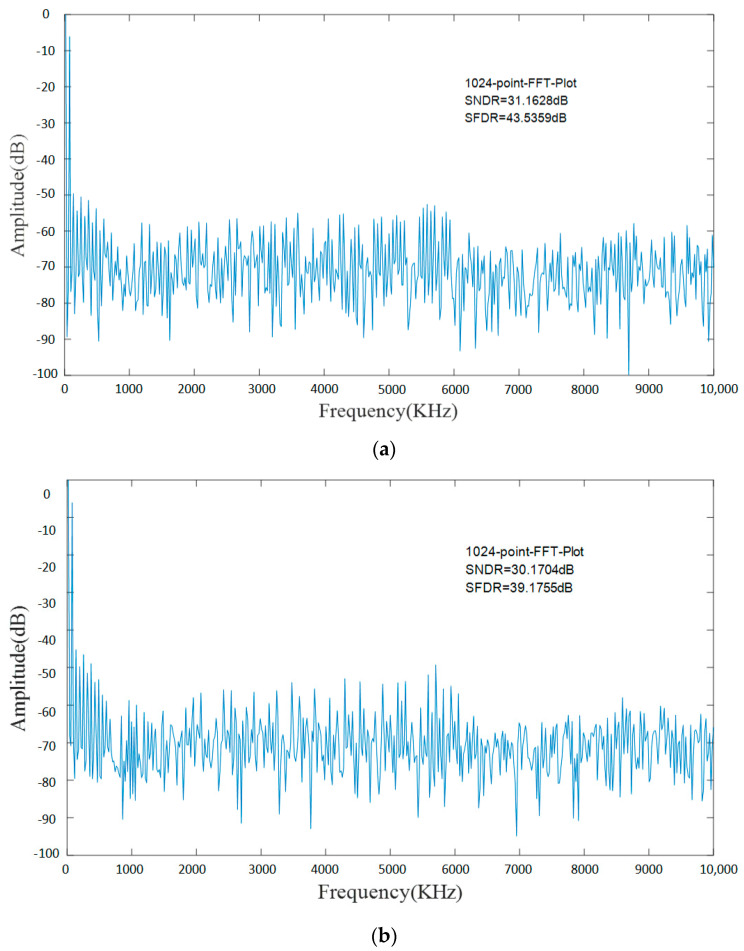
Functional simulation of TDC. (**a**) Odd−line TDC spectrum. (**b**) Even−line TDC spectrum.

In order to investigate the dynamic behavior of the proposed TDC, a full-scale sinusoidal input signal at 58.594 kHz was applied to the circuit and the analog equivalent of the output digital codes and its spectrum were calculated and are depicted in Figure 10. Since the logic circuit before the odd-line and even-line TDC is slightly different, and therefore has a different spectrum, it can be observed that the signal-to-noise and distortion ratio (SNDR) of the odd-line TDC is 31.1628 dB, resulting in an effective number of bits (ENOB) at 4.88 bits. In addition, the spurious free dynamic range (SFDR) of the circuit is 43.5359 dB. The even-line TDC has a signal-to-noise and distortion ratio (SNDR) of 30.1704 dB, resulting in an effective number of bits (ENOB) at 4.72 bits, and a spurious-free dynamic range (SFDR) of 39.1755 dB.

Finally, Table 1 compares the performance of the proposed low-power structure with the other works. In Ref. [12], the value of the new sample signal is encoded according to the difference between the previous signal and the new sample signal, and the continuity of the analog signal in time is utilized to reduce the power consumption of the circuit. The proposed TDC uses the continuity of the analog signal in space to reduce the counting power consumption of a TDC and has a lower optimization coefficient FOM. In addition, the method of quantizing two rows of pixel output at the same time is more suitable for high-speed image sensor applications. Compared with a TDC in two-step SS ADC applied to a CMOS image sensor in Ref. [14], the proposed TDC has a lower power consumption and FOM. A two-step TDC based on a time amplifier was proposed by Ref. [15], in which only one time amplifier is activated at each conversion stage, so that the TDC has a better system energy efficiency. The FOM for the proposed TDC has obvious advantages compared with it.

## 5. Conclusions

In this work, a low-power TDC structure is proposed, which is mainly used for high-speed SS ADC of CMOS image sensors. The proposed TDC utilizes the continuity of analog signals between rows of image sensor pixels and quantifies the difference between the two adjacent rows of pixel outputs by adjusting the *start* and *stop* signal inputs of the all-digital TDC to reduce the number of flips of the D flip-flop on the voltage-controlled delay line.

Compared with a traditional TDC, the quantization method of a two-row TDC can greatly relieve the pressure of high-speed image sensor row selection time and meet the needs of high-speed design. At the same time, it quantifies the difference between two rows of pixel signals rather than the complete pixel signal, so its counting power consumption is greatly reduced; when the quantization bit reaches five bits, its power consumption reduction level can reach more than 30%. The proposed TDC can achieve a FOM of 104.7–162.3 fJ/step with a significant reduction in the counting power consumption.

## Figures and Tables

**Table 1:** Performance comparison of the proposed TDC structure with the other works.

	[12]	[14]	[15]	This Work
CMOS process	65 nm	0.11 μm	0.18 μm	0.11 μm
Number of bits	8	12	8	5
Sampling rate (MS/s)	0.2	0.5	30	20
Power (μW)	10	187	1100	60–93
ENOB (bits)	7.52	10.68	6.07	4.72
FOM * (fJ/step)	275.0	182.6	540.0	104.7–162.3

* FOM = (Power × 1 Row Time)/2^ENOB^.

## Data Availability

This paper is a study of TDC structure, there is no data set, all simulation results are based on the circuit model, the input signal voltage can be provided to achieve, simulation results have been shown.
